# Synchronous Papillary Thyroid Microcarcinoma and Chronic Lymphocytic Leukemia: A Rare Interdisciplinary Case

**DOI:** 10.7759/cureus.107194

**Published:** 2026-04-16

**Authors:** Roman Farnin, Halil Altindag, Robert Lienenlüke, Antonia Hammer, Lula Gebrehiwot, Johanna Engler, Julian Mittermeier, Christian Vorländer

**Affiliations:** 1 Endocrine Surgery, Bürgerhospital, Frankfurt am Main, DEU

**Keywords:** cervical lymphadenopathy, chronic lymphocytic leukemia, differential diagnosis, immunohistochemistry, papillary thyroid microcarcinoma, synchronous malignancy

## Abstract

Papillary thyroid microcarcinoma is a common incidental finding due to the widespread use of high-resolution ultrasound imaging. Cervical lymphadenopathy, however, may have diverse etiologies, including metastatic disease and hematologic malignancies. We report a rare case of synchronous papillary thyroid microcarcinoma and chronic lymphocytic leukemia (CLL) presenting with cervical lymphadenopathy. The patient described in this case report underwent total thyroidectomy with left lateral neck dissection due to suspicious bilateral thyroid nodules and enlarged cervical lymph nodes. Histopathological examination confirmed papillary microcarcinoma, while additional immunohistochemical analysis of lymph nodes revealed a low-grade B-cell non-Hodgkin lymphoma consistent with CLL. This case highlights the importance of considering alternative diagnoses in patients with lymphadenopathy and emphasizes the role of thorough histopathological evaluation in guiding appropriate management.

## Introduction

In recent years, there has been an apparent increase in the incidence of thyroid cancer worldwide. This trend is particularly observed in countries with widespread access to high-resolution ultrasonography, including South Korea, Japan, Germany, and the United States [1-3]. It is largely attributed to increased detection of small papillary carcinomas due to the widespread use of advanced imaging techniques, rather than a true increase in disease occurrence [1].

According to data from the Surveillance, Epidemiology, and End Results (SEER) program, the age-adjusted incidence of papillary thyroid carcinoma (PTC) is approximately 14-16 cases per 100,000 population per year, with a higher proportion observed in women [4]. Papillary microcarcinomas now account for a substantial proportion of newly diagnosed cases and are often detected incidentally during imaging procedures [5]. This development raises new questions about the clinical relevance of small thyroid carcinomas and their distinction from indolent lymphatic disorders-particularly in the presence of unclear cervical lymphadenopathy. Such lymphadenopathy can, from a differential diagnostic perspective, indicate not only inflammatory processes or metastatic involvement from a thyroid carcinoma, but may also be a manifestation of a hematologic neoplasm such as chronic lymphocytic leukemia (CLL) [6,7]. Early identification is clinically important because the choice of treatment, including the scope of surgery, the use of medication, and the follow-up strategy, can differ significantly depending on the underlying cause.

CLL is the most common form of leukemia in adults, with an age-adjusted incidence rate of 4.9 cases per 100,000 individuals per year. The median age at diagnosis is approximately 70 years, and only 9.1% of patients are younger than 45. Men are affected more frequently than women, with a male-to-female ratio of about 1.9 to 1, a disparity that remains consistent across all ethnic groups. It is estimated that approximately 0.6% of the population will develop CLL during their lifetime. Pathophysiologically, CLL is characterized by the clonal proliferation and accumulation of mature, typically CD5-positive B cells in the peripheral blood, bone marrow, lymph nodes, and spleen [7].

The coexistence of CLL and PTC is extremely rare [8-10]. The simultaneous detection of both conditions requires an interdisciplinary approach and precise surgical as well as histopathological evaluation. The present case exemplifies the diagnostic and therapeutic challenges associated with this uncommon clinical constellation.

Whether the coexistence of papillary thyroid microcarcinoma and CLL represents a coincidental finding or reflects a potential underlying immunological association remains unclear and contributes to the diagnostic complexity of such cases.

## Case presentation

A 50-year-old female patient initially presented for evaluation of a multinodular goiter that had been known for several years. She reported unintentional weight loss without accompanying B symptoms. There was no relevant family history of thyroid or hematologic malignancy. The patient was not receiving any thyroid-specific medication and reported only occasional use of cholecalciferol.

Laboratory findings are summarized in Table 1. 

**Table 1:** Summary of laboratory findings FT3: Free triiodothyronine; FT4: Free thyroxine; TSH: Thyroid-stimulating hormone; TPOAb: Thyroid peroxidase antibodies; TRAb: TSH receptor antibodies; MCV: Mean corpuscular volume; MCH: Mean corpuscular hemoglobin

Parameter	Value	Unit	Reference range
Calcium	2.32	mmol/L	2.15–2.50
25-hydroxyvitamin D	89	nmol/L	75–100
FT3	3.9	pmol/L	3.1–6.8
FT4	13	pg/mL	9.2–16.8
TSH	0.76	mIU/L	0.27–4.2
TPOAb	Negative	-	Negative
TRAb	<0.8	IU/L	<1.75
Calcitonin	7.8	pg/mL	<10
MCV	95.9	fL	80–96
MCH	30.1	pg	28–33

Additionally, the complete blood count indicated a macrocytic profile with elevated mean corpuscular volume (MCV) and mean corpuscular hemoglobin (MCH), as shown in Table 1, which may represent a subtle indicator of an underlying hematologic disorder. Other parameters were within normal limits.

Thyroid function was euthyroid, and the tumor marker calcitonin was within the normal range. Initial thyroid scintigraphy revealed reduced uptake in the right centrolateral and left centrocaudal regions. Subsequent ultrasound examination demonstrated a suspicious hypoechoic nodule in the right thyroid lobe, classified as Thyroid Imaging Reporting and Data System (TIRADS) 4c. Fine-needle aspiration (FNA) performed thereafter on a nodule in the right thyroid lobe yielded benign cytology according to the Bethesda System for Reporting Thyroid Cytopathology (Bethesda II).

**Figure 1:**
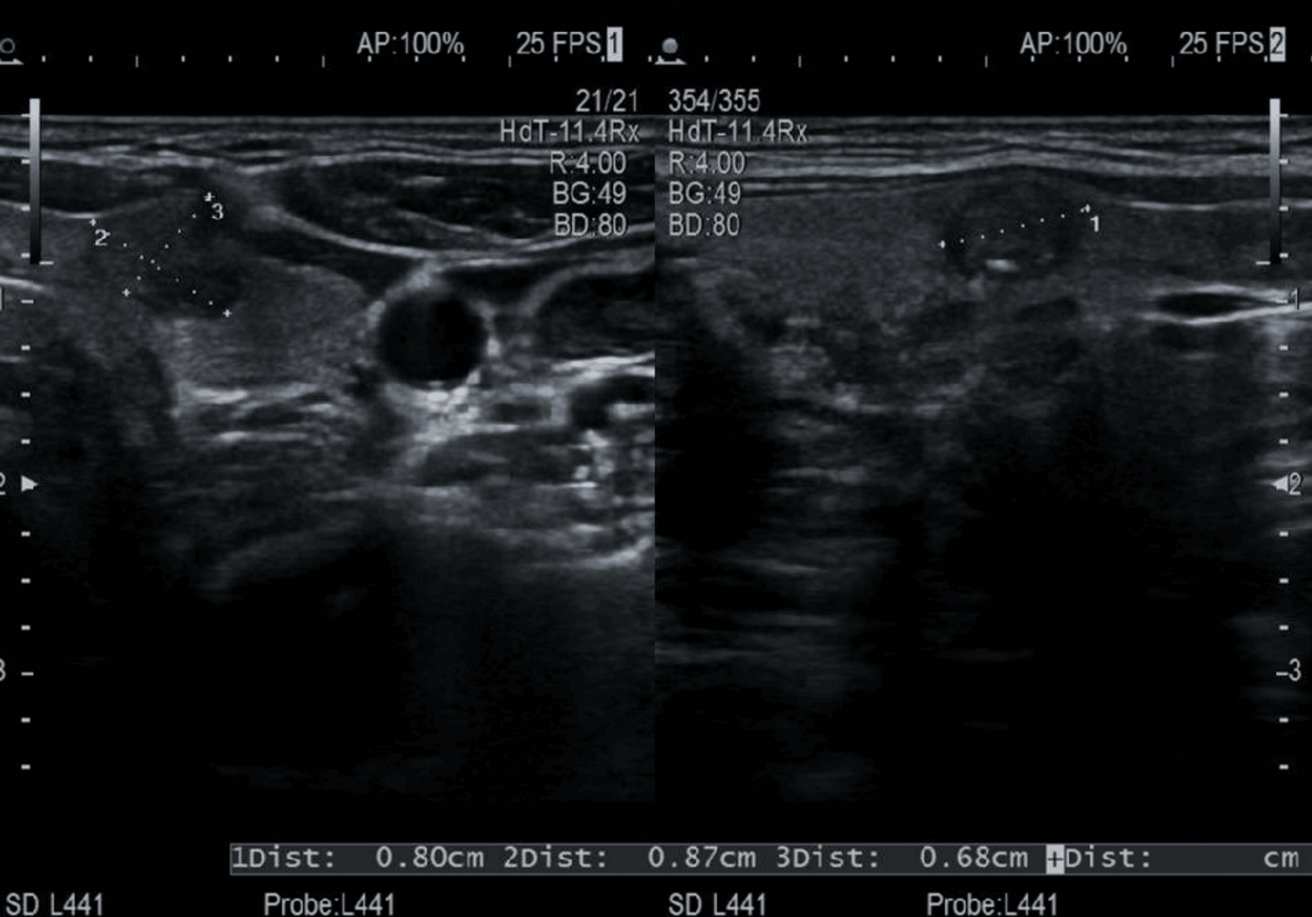
Transverse thyroid ultrasound Transverse B-mode ultrasound image showing multiple hypoechoic nodules in the left thyroid lobe, the largest measuring approximately 0.87 × 0.80 × 0.68 cm.

**Figure 2:**
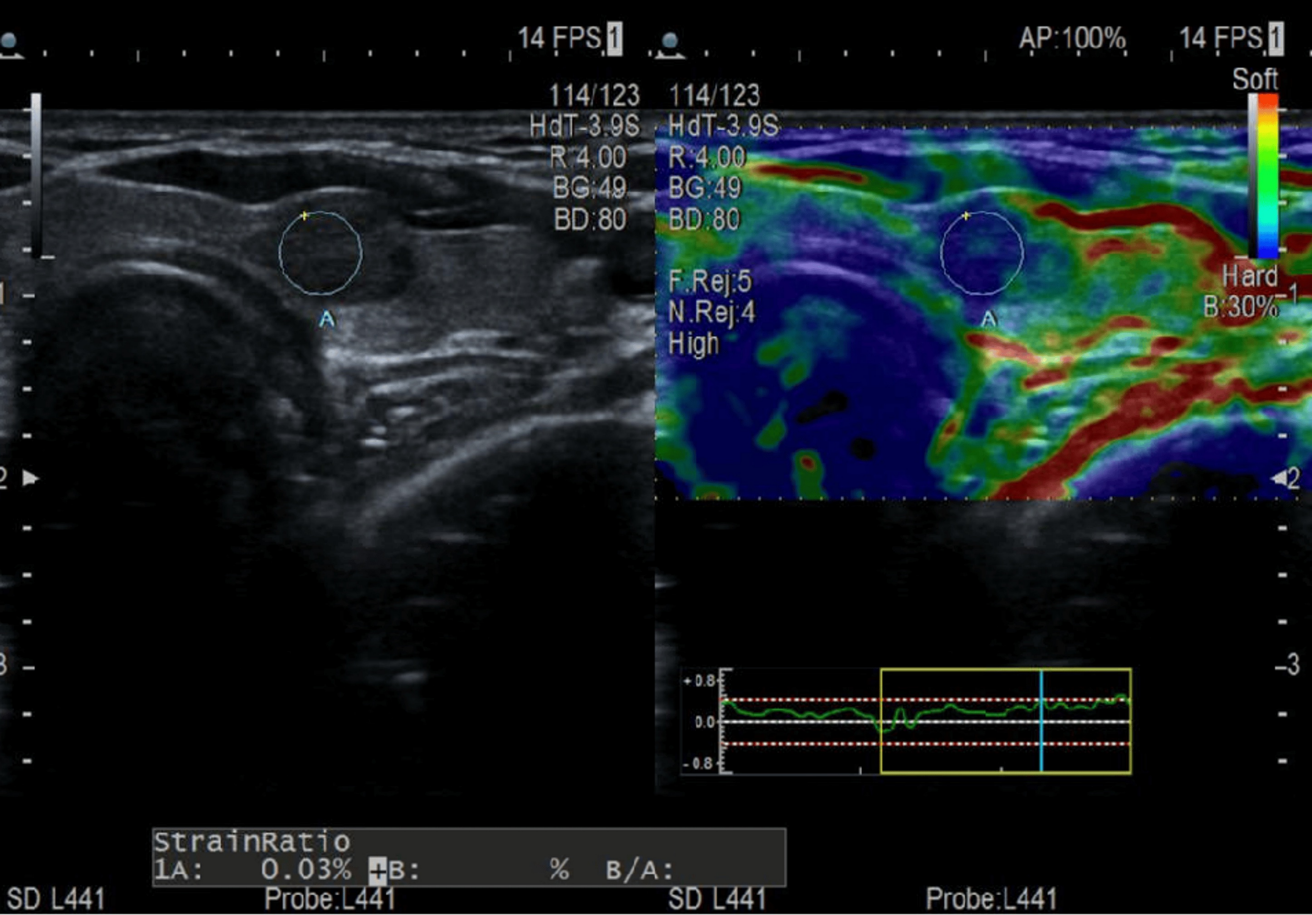
Thyroid ultrasound with elastography B-mode ultrasound with corresponding elastography image showing a suspicious left thyroid nodule with high stiffness (strain ratio: 0.03%), consistent with TIRADS 4c. TIRADS: Thyroid Imaging Reporting and Data System

Following these findings, the patient reported newly developed and progressively enlarging lymph nodes in the left lateral cervical region. Additional ultrasound examination revealed several hypoechoic nodules in the left thyroid lobe, with the largest measuring approximately 0.87 × 0.80 × 0.68 cm (Figure 1). One of the nodules exhibited increased stiffness on elastography with a strain ratio of 0.03%, corresponding to a high suspicion for malignancy (Figure 2).

**Figure 3:**
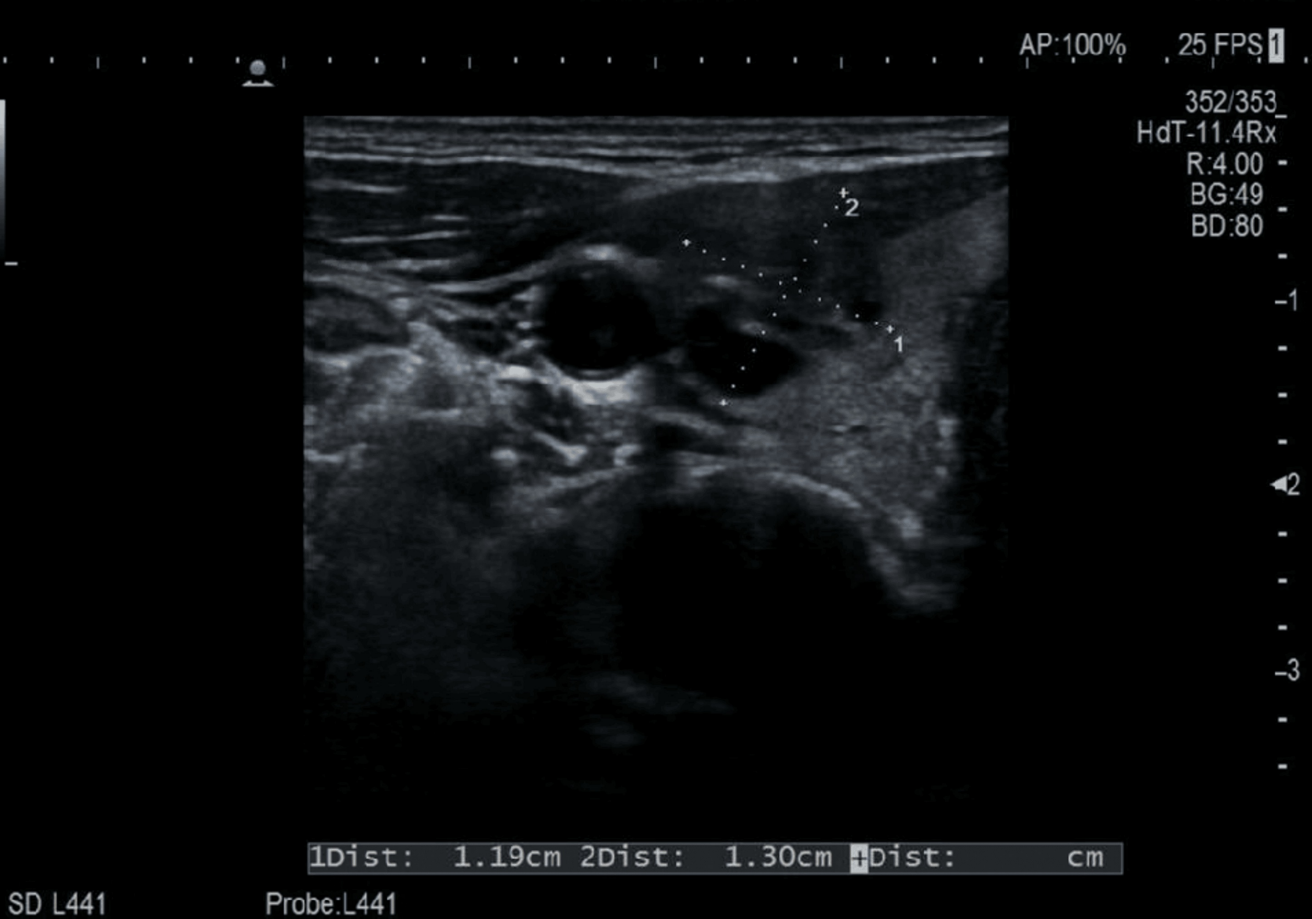
Cervical lymph node ultrasound Transverse ultrasound image of a left-sided cervical lymph node (level IV) demonstrating a hypoechoic cortex and central echogenic hilum. Dimensions: 1.19 × 1.30 cm.

**Figure 4:**
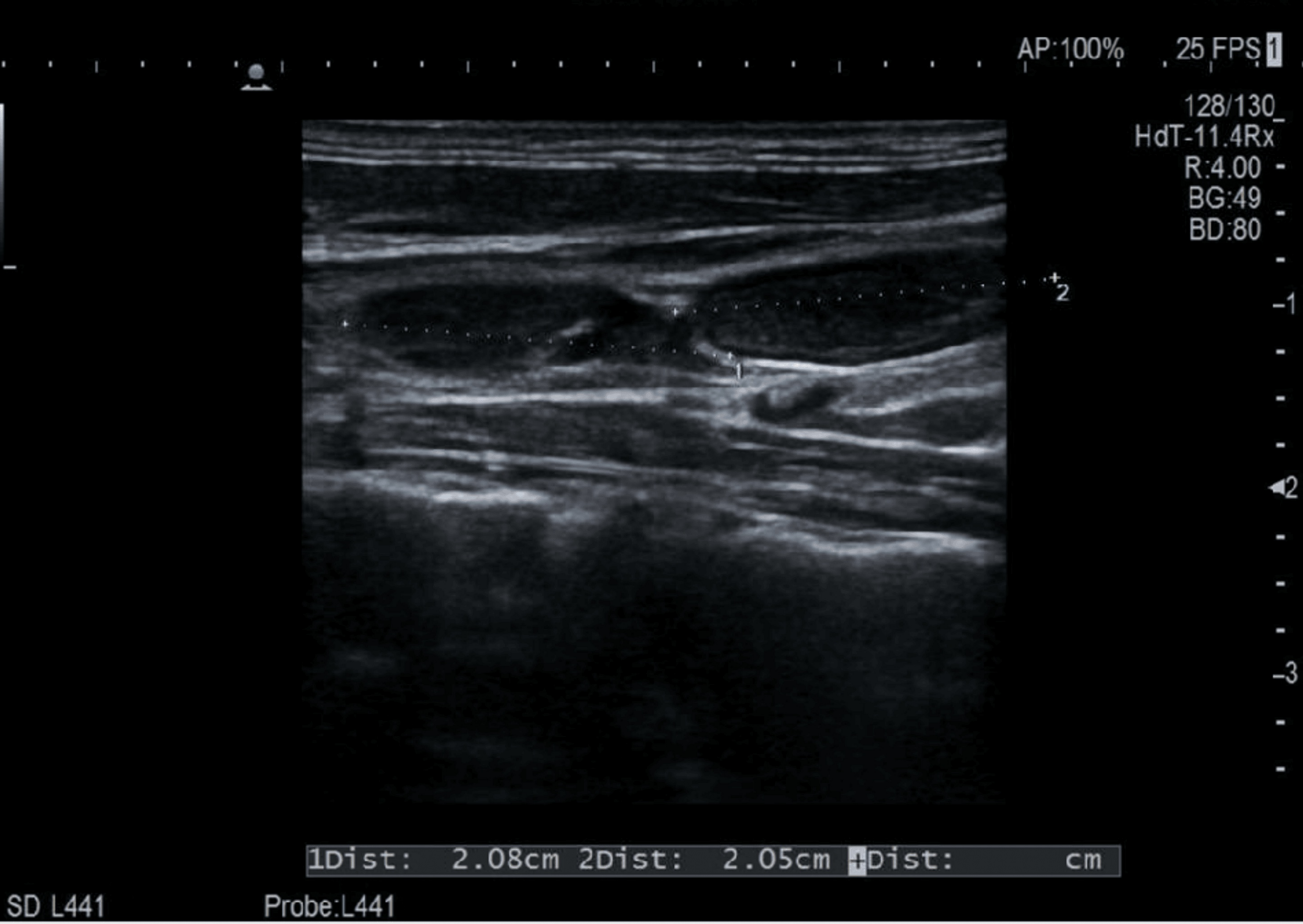
Longitudinal cervical lymph node ultrasound Longitudinal ultrasound image of a left-sided cervical lymph node (level IV) measuring approximately 2.08 × 2.05 cm.

These nodules were classified as TIRADS 4c and were located ipsilateral to the cervical pathological lymphadenopathy. Cervical ultrasound demonstrated several enlarged lymph nodes in the left level IV region, raising suspicion for pathological lymphadenopathy (Figures 3, 4).

A total thyroidectomy with left selective neck dissection was performed due to suspicious cervical lymphadenopathy and a preoperative suspicion of metastatic thyroid carcinoma. The lymph node dissection was undertaken with both diagnostic and therapeutic intent. Intraoperative frozen section analysis was not performed. Histopathological analysis confirmed the diagnosis post-operatively and revealed a 7 mm papillary microcarcinoma in the left thyroid lobe, along with bilateral nodular goiter. According to the Tumor-Node-Metastasis (TNM) classification (8th edition, 2020), the tumor was staged as pT1a, pN0 (0/9), L0, V0, Pn0, R0.

In addition, the lymph nodes showed B-cell-dominant infiltrates. Immunohistochemical analysis demonstrated positivity for CD20, CD23, BCL2, and MNDA. Partial weak staining for CD5 was observed, and T cells were positive for CD3 and LEF1. Scattered cells were positive for cyclin D1. The proliferation index (Ki-67) was elevated in the proliferation centers.

At the time of surgery, CLL was neither known nor clinically suspected. The diagnosis was made postoperatively based on the histological examination of the excised lymph nodes. The patient was referred for further hematologic evaluation. Given the tumor profile, radioiodine therapy was not indicated. A follow-up plan with semiannual monitoring was established.

## Discussion

This case highlights several important diagnostic challenges. Notably, FNA cytology was performed on a nodule in the right thyroid lobe and was classified as Bethesda II (benign). However, final surgical pathology revealed papillary thyroid microcarcinoma in the contralateral (left) lobe. This discrepancy is likely explained by sampling of a different, non-malignant nodule, while the suspicious lesion in the left lobe was not initially biopsied. In the setting of multinodular goiter, this underscores the potential for sampling error and highlights the importance of carefully correlating imaging findings, especially suspicious ultrasound features and associated lymphadenopathy, with the selection of biopsy targets.

The decision to proceed with total thyroidectomy despite benign cytology was based on the overall context of multiple risk factors: bilateral TIRADS 4c nodules, multinodular goiter, and newly developed cervical lymphadenopathy. This constellation justified an extended surgical approach, in line with the American Thyroid Association guidelines in effect at the time of surgery, as well as recommendations from the European Society for Medical Oncology regarding suspicious multilocular thyroid lesions with abnormal lymph node involvement [11,12]. 

Cervical lymphadenopathy poses a diagnostic challenge. In addition to metastatic thyroid carcinoma, infectious, autoimmune, and lymphoproliferative disorders must be considered. The initial histopathological evaluation did not reveal evidence of CLL. The definitive diagnosis was established only in the final pathology report following comprehensive histopathological assessment, with immunohistochemical analysis confirming a low-grade B-cell non-Hodgkin lymphoma of the CLL type. The immunophenotype was characterized by the expression of CD20, CD23, BCL2, as well as partial positivity for CD5 and LEF1, along with an elevated proliferation index (Ki-67).

The simultaneous diagnosis of PTC and CLL has only rarely been described in the literature. Case reports have described similar findings, including instances where both entities occurred concurrently within the same lymph node [8-10]. In a previous analysis, hematological neoplasms were reported to be incidentally found in less than 1% of thyroidectomy specimens [6]. In contrast to primary thyroid lymphomas, which are often associated with Hashimoto’s thyroiditis, CLL typically represents secondary involvement of cervical lymph nodes. Whether the concurrent diagnosis represents a purely coincidental finding or an immunologically mediated association remains uncertain [7,13]. 

As shown in prospective studies, papillary microcarcinomas often follow an indolent course, making active surveillance an increasingly adopted management strategy [2,14]. However, these conservative approaches contrast with cases involving atypical features such as persistent lymphadenopathy, which - as in our case -may conceal a hematologic malignancy such as CLL in the differential diagnosis. An interdisciplinary approach, including surgical, endocrinological, and hematological consultation, was critical for early identification and targeted management. Following multidisciplinary tumor board discussion, the patient was referred to a specialized hemato-oncology center for further staging and management of CLL.

## Conclusions

This case highlights the importance of a comprehensive and structured approach in the evaluation of thyroid nodules accompanied by atypical lymphadenopathy. Despite benign cytology, the presence of suspicious nodules on imaging and unclear cervical lymph nodes led to an extended surgical indication, which, in retrospect, was essential for the simultaneous detection of two malignant conditions.

The concurrent diagnosis of PTC and a low-grade B-cell lymphoma (CLL type) highlights the limitations of traditional organ-centered diagnostic paradigms. It demonstrates that seemingly benign thyroid disorders may conceal clinically significant neoplasms. These findings underscore the value of precise imaging, critical interpretation of cytological results, and thorough histopathological and immunohistochemical assessment. A coordinated multidisciplinary management strategy is essential for appropriate treatment and follow-up. In cases of thyroid nodules with atypical lymphadenopathy, a hematologic neoplasm should always be considered in the differential diagnosis.
